# Hybrid closed wedge high tibial osteotomy is associated with short-term arthroscopic changes in the patellofemoral joint

**DOI:** 10.1186/s12891-026-09779-w

**Published:** 2026-04-14

**Authors:** Mikio Shioda, Ryohei Takeuchi, Woon-Hwa Jung, Hiroyuki Ishikawa, Katsunari Osawa, Yuichiro Yamaguchi, Umito Kuwashima, Hideyuki Koga

**Affiliations:** 1 Clinical Center for Sports Medicine and Sports Dentistry, Institute of Science Tokyo, Tokyo, Japan; 2 Joint Surgery Center, Yokohama Sekishinkai Hospital, 21-1 Toyooka-chyo, Tsurumi-ku, Yokohama-Shi, Kanagawa, 230-0062 Japan; 3 Department of Orthopedic Surgery, Dokkyo Medical University Saitama Medical Center, Saitama, Japan; 4 Department of Orthopaedic Surgery, Murup Hospital, Jungang-dong, Masanhappo-gu, Changwon-si, Republic of Korea; 5 Department of Joint Surgery Center, Yokosuka Municipal Hospital, Yokosuka, Japan; 6 Department of Orthopaedic Surgery, Tokyo Women’s Medical University, Tokyo, Japan; 7 Department of Joint Surgery and Sports Medicine, Institute of Science Tokyo, Tokyo, Japan

**Keywords:** Arthroscopy, Cartilage, Hybrid closed wedge high tibial osteotomy, Osteoarthritis, Patellofemoral joint

## Abstract

**Background:**

Hybrid closed wedge high tibial osteotomy (HCWHTO) has been reported to influence patellofemoral (PF) joint status; however, macroscopic cartilage changes remain insufficiently documented. This study aimed to evaluate 1-year arthroscopic changes in trochlear cartilage after HCWHTO and to examine their association with 2-year clinical outcomes.

**Methods:**

Between July 2011 and May 2016, 77 patients (102 knees) underwent primary HCWHTO at our institution. Of these, 61 patients (81 knees) had complete second-look arthroscopy at approximately one year postoperatively and clinical and radiographic follow-up at a minimum of two years. These cases constituted the final analysis cohort. Preoperative radiographic severity was assessed using the Kellgren–Lawrence classification. All included knees were classified as grade 3 or 4. Cartilage status was assessed arthroscopically at approximately 1 year postoperatively using the International Cartilage Repair Society (ICRS) grading system. All analyses were performed on a per-knee basis.

**Results:**

All included knees were classified as Kellgren–Lawrence grade 3 or 4. At 1-year follow-up, 37.0% of knees demonstrated macroscopic cartilage surface improvement in the trochlear groove, whereas deterioration was observed in 6.2%. Although the median ICRS grade of the trochlea remained unchanged, the distribution shifted toward lower grades (*p* < 0.01). The medial patellar facet showed a slight improvement in median ICRS grade, while the lateral facet remained largely unchanged. All J-KOOS subscale scores improved significantly at 2 years postoperatively (*p* < 0.001). No significant correlation was observed between arthroscopic cartilage changes and improvements in clinical outcomes.

**Conclusions:**

In knees with complete 2-year follow-up, HCWHTO was associated with macroscopic cartilage surface changes, including redistribution toward lower ICRS grades in the trochlea at 1 year after surgery. However, these changes were not significantly correlated with clinical outcomes at 2 years postoperatively.

**Supplementary Information:**

The online version contains supplementary material available at 10.1186/s12891-026-09779-w.

## Introduction

High tibial osteotomy (HTO) is an established and effective surgical treatment for medial compartment osteoarthritis of the knee. Conventional closed wedge high tibial osteotomy (CWHTO) is particularly suitable for cases requiring large correction angles and has demonstrated favorable long-term outcomes over 20 years in multiple studies [1]. However, this procedure is associated with several disadvantages, including removal of a large bone wedge, increased lateral offset, and delayed progression to full weight bearing [2]. To overcome these limitations, hybrid closed wedge high tibial osteotomy (HCWHTO) was developed [2]. In this technique, the hinge point is shifted slightly from the medial cortex toward the lateral side, typically to one-third or one-quarter of the osteotomy line, allowing partial preservation of medial bone. Recent studies have reported favorable clinical outcomes and high rates of return to sports following HCWHTO [3]. In addition, improvements in patellofemoral joint osteoarthritis (PFOA) have been observed clinically after this procedure; however, objective evaluations of patellofemoral (PF) joint changes remain limited.

Open wedge high tibial osteotomy (OWHTO) has gained widespread acceptance because of its technical simplicity and rigid fixation, which facilitate accurate correction according to the preoperative plan. Nevertheless, recent systematic reviews and studies have consistently reported patellar height reduction and increased PF contact pressure following OWHTO [4–7].

Consequently, careful consideration is required when selecting OWHTO for patients with medial compartment osteoarthritis accompanied by PFOA. Open wedge distal tuberosity osteotomy (DTO) has been introduced as an alternative technique to mitigate the negative effects of OWHTO on the PF joint [8, 9]. Although arthroscopic evaluations have suggested a lower incidence of PFOA compared with OWHTO, the long-term clinical outcomes of DTO remain unclear [10, 11]. Furthermore, DTO may not provide sufficient quadriceps tension and mechanical stability to promote reliable bone union at the osteotomy site [12]. Despite the potential advantages of HCWHTO, only a limited number of studies have investigated its impact on the PF joint using direct arthroscopic assessment. The purpose of this study was to descriptively characterize short-term arthroscopic changes in the PF joint before and after HCWHTO. This study was conducted as an exploratory descriptive case series without a comparative cohort. We hypothesized that HCWHTO would not adversely affect short-term PF cartilage status as assessed by second-look arthroscopy. Clarifying the short-term arthroscopic response of the PF joint following HCWHTO may provide clinically relevant information to support surgical decision-making in patients with medial compartment osteoarthritis accompanied by PFOA.

## Materials and methods

### Patient selection

**Fig. 1:**
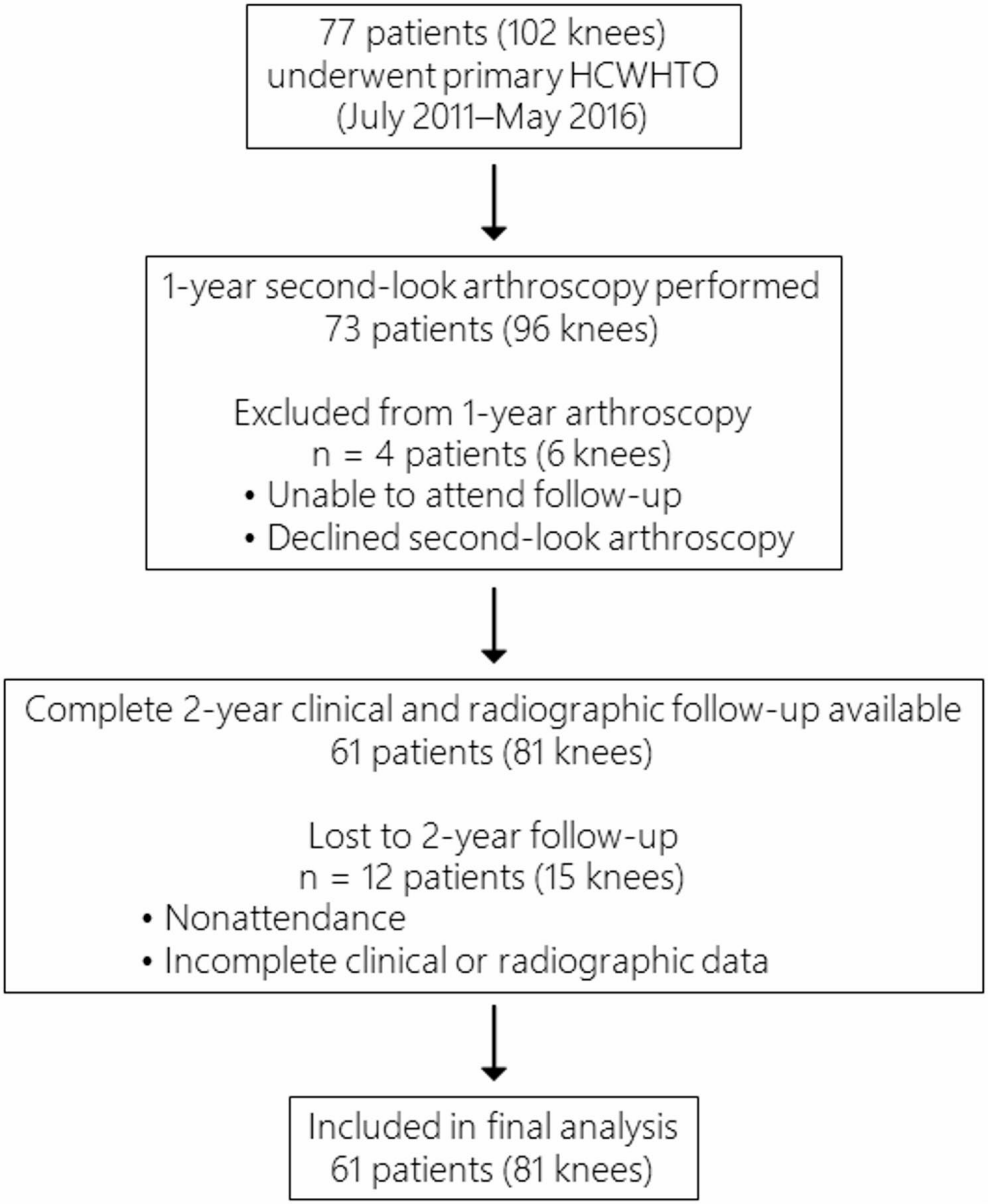
Flow diagram of patient enrollment and follow-up. Between July 2011 and May 2016, 77 patients (102 knees) underwent primary hybrid closed wedge high tibial osteotomy (HCWHTO). Of these, 73 patients (96 knees) underwent second-look arthroscopy at approximately 1 year postoperatively. At a minimum of 2 years postoperatively, complete clinical and radiographic follow-up data were available for 61 patients (81 knees), who constituted the final analysis cohort. Analyses were performed on a per-knee basis

This retrospective case series was approved by the Institutional Review Board of Yokosuka Municipal Hospital (Approval No. Shimin-Rinri-Hatsu 30–2), and written informed consent was obtained from all participants. Between July 2011 and May 2016, a total of 77 patients (102 knees) underwent primary HCWHTO at our institution. Of these, 73 patients (96 knees) underwent second-look arthroscopy at approximately one year postoperatively. At a minimum of two years postoperatively, complete clinical and radiographic follow-up data were available for 61 patients (81 knees). Only patients with both second-look arthroscopy and complete 2-year clinical follow-up were included in the final analysis. 61 patients (81 knees) constituted the analysis cohort (Fig. 1). All analyses were performed on a per-knee basis.

### Indications for surgery

General indications for HTO include symptomatic medial compartment osteoarthritis refractory to conservative treatment and varus malalignment. During the study period, HCWHTO was selected according to the surgeon’s standard clinical practice, particularly in cases with coexisting PF pathology or when a relatively large correction angle was required. The present analysis includes only cases treated with HCWHTO.

### Surgical procedure

All procedures were performed by three experienced orthopedic surgeons using a standardized HCWHTO technique as previously described by Takeuchi et al. [2]. Diagnostic arthroscopy was routinely performed prior to osteotomy, and partial meniscectomy was conducted when irreparable degenerative meniscal tears were identified. The osteotomy was fixed using a lateral HTO plate or a proximal lateral tibial plate. The target postoperative percentage mechanical axis deviation (%MAD) was set at 62.5% [13]. During the study period, two types of lateral fixation plates were used. Until December 2015, a proximal lateral tibial plate (DePuy Synthes, Oberdorf, Switzerland) was used. The Olympus lateral HTO plate (Olympus Terumo Biomaterials, Tokyo, Japan) was introduced in January 2016 and was used in 11 patients (14 knees) included in the final analysis cohort. Implant selection was determined chronologically according to the time period without specific patient-related criteria. No formal comparison according to implant type was performed because the implant change was time-dependent and the number of cases treated with the newer plate was limited. Implant removal was routinely performed approximately one year after the initial surgery. At the time of plate removal, second-look arthroscopy and clinical evaluations were conducted after obtaining renewed written informed consent.

### Postoperative rehabilitation

Postoperative rehabilitation began on the first postoperative day and included range-of-motion exercises using continuous passive motion devices and standing exercises. Active range-of-motion and muscle-strengthening exercises were introduced concurrently.

Partial weight-bearing with a walker was initiated on postoperative day two, depending on patient tolerance. Full weight-bearing was permitted from the second postoperative week.

### Evaluation

Clinical outcomes were assessed using the Japanese version of the Knee injury and Osteoarthritis Outcome Score (J-KOOS), visual analog scale (VAS) for pain, and knee range of motion. Clinical evaluations were performed preoperatively and at a minimum of 2 years postoperatively [14, 15].

### Radiographic evaluation (2-year follow-up)

**Fig. 2:**
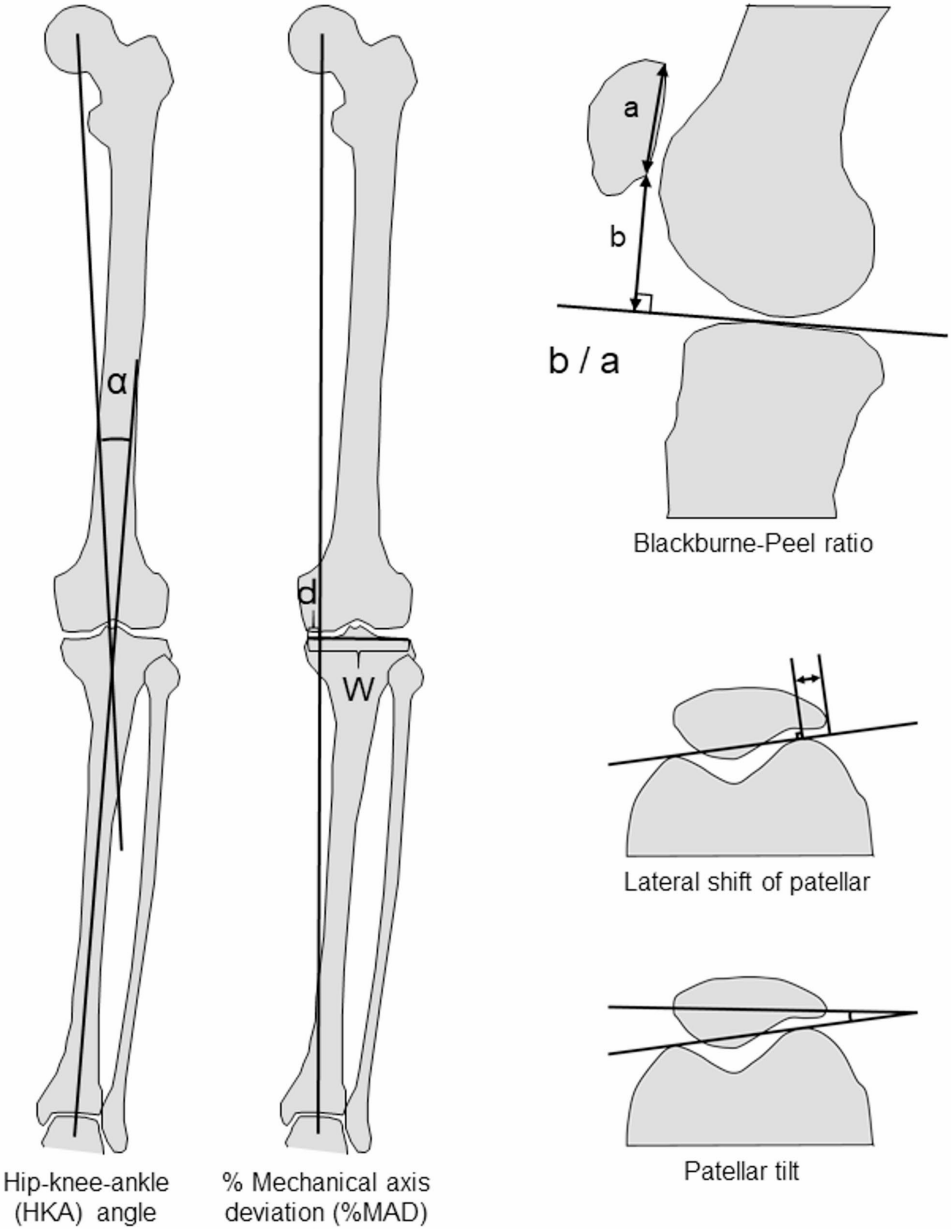
Evaluation of radiograph. **A **The hip–knee–ankle (HKA) angle was defined as the angle (α) between the mechanical axes of the femur and tibia on full-length standing radiographs. **B** Percentage mechanical axis deviation (%MAD) was calculated as d/W × 100, where d represents the distance from the medial edge of the proximal tibia to the point where the mechanical axis (line from the center of the femoral head to the center of the ankle) intersects the tibial surface, and W represents the total width of the proximal tibia. **C** The Blackburne–Peel ratio was evaluated using weight-bearing lateral knee radiographs obtained at approximately 30° of flexion. **D** Lateral shift of the patella was measured on skyline radiographs and defined as the distance between the highest point of the lateral femoral condyle and the intersection of (i) a line connecting the highest points of both femoral condyles and (ii) a perpendicular line drawn from the lateral edge of the patella. **E** Patellar tilt was measured on skyline radiographs and defined as the angle between a line connecting the highest points of the femoral condyles and a line intersecting the widest bony diameter of the patella. This figure was created by the authors and has not been previously published

Radiographic evaluations were based on preoperative and approximately 2-year postoperative radiographs. The Hip–Knee–Ankle (HKA) angle, %MAD, Blackburne–Peel ratio, patellar tilt, and lateral shift of the patella were evaluated using digital software (Plissimo; Konia, Tokyo, Japan), as illustrated in Fig. 2. Radiographic measurements were performed independently by two orthopedic surgeons who were blinded to each other’s results and to the clinical data. To assess interobserver reliability, 10 cases were randomly selected from the study cohort. For each case, both preoperative and postoperative radiographic parameters were measured independently by both observers. Interobserver reliability was evaluated using intraclass correlation coefficients (ICC) based on a two-way random-effects model for absolute agreement (ICC [1, 2]). ICC values were calculated separately for preoperative and postoperative measurements. In addition, combined analyses including both preoperative and postoperative values were performed as supplementary analyses. 95% confidence intervals (95% CIs) were computed for all ICC estimates. Intraobserver reliability was not formally assessed, as each parameter was measured once per observer within the retrospective study design; however, standardized digital measurement software and predefined anatomical landmarks were used to minimize variability.

### Arthroscopic evaluation (1-year follow-up)

**Fig. 3:**
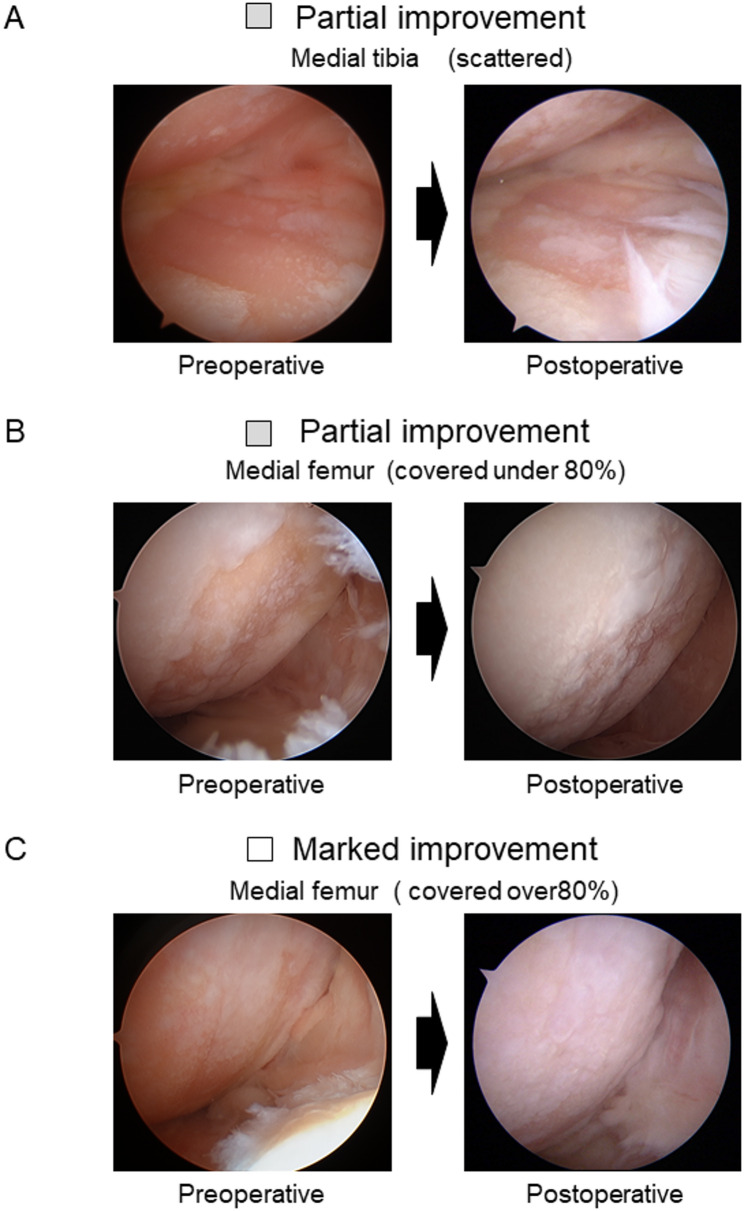
Representative paired arthroscopic images demonstrating marked and partial improvement in macroscopic cartilage surface appearance from baseline to 1-year follow-up. Representative arthroscopic findings demonstrating macroscopic cartilage surface improvement. **A** Partial improvement in the medial tibial plateau with scattered repair-like tissue. **B** Partial improvement in the medial femoral condyle with broad but incomplete surface coverage. **C **Marked improvement in the medial femoral condyle with near-complete surface coverage of the defect

Arthroscopic evaluations were performed using intraoperative findings and approximately 1-year postoperative follow-up findings. For absolute evaluation, the International Cartilage Repair Society (ICRS) grading system was used, and the worst grade within each region of interest was recorded. Relative evaluation was performed using a four-category scale: marked improvement, partial improvement, no change, and deterioration. “Marked improvement” was defined as > 80% surface coverage of the defect with repair-like tissue appearance, whereas “partial improvement” was defined as ≤ 80% surface coverage or patchy surface coverage (Fig. 3). Two independent orthopedic surgeons who were not involved in the surgical procedures evaluated all arthroscopic findings. When discrepancies occurred, the lower score was adopted for analysis. Seven articular regions were assessed: medial and lateral patellar facets, femoral trochlear groove, medial and lateral femoral condyles, and medial and lateral tibial plateaus.

### Statistical analysis

Normality of continuous variables was assessed by visual inspection of histograms. Continuous radiographic parameters were analyzed using paired t-tests and are presented as mean ± standard deviation (SD). The primary structural outcome was the change in trochlear cartilage status assessed using the ICRS grading system. Because ICRS grades are ordinal variables, results are presented as median and interquartile range (IQR). Preoperative and postoperative ICRS grades were compared using the Wilcoxon signed-rank test. The magnitude of change was quantified using the effect size r (r = Z/√N). To explore the association between clinical improvement and arthroscopic cartilage status, Spearman’s rank correlation coefficient was used to assess the relationship between changes in each of the five J-KOOS subscales (Pain, Symptoms, ADL, Sport/Rec, and QOL; 24 months minus preoperative) and changes in trochlear ICRS grade (postoperative minus preoperative). In addition, changes in each J-KOOS subscale were compared between knees with macroscopic arthroscopic cartilage surface improvement (marked/partial improvement) and those without improvement (no change/deteriorated) using the Mann–Whitney U test. A two-sided p value < 0.05 was considered statistically significant. For the primary clinical outcome (J-KOOS), effect sizes for paired comparisons were expressed as Cohen’s dz. A post hoc power analysis was performed based on the observed effect size of the paired t-test (Cohen’s dz = 1.12), indicating a large effect. However, interpretation of the results was primarily based on effect sizes and 95% confidence intervals rather than post hoc power estimates. Statistical analyses were performed using IBM SPSS Statistics (version 30.0.0.0 (171); IBM Corp., Armonk, NY, USA).

## Results

### Patient characteristics

61 patients (81 knees) were included in the final analysis cohort. All knees were classified as Kellgren–Lawrence grade 3 (*n* = 25) or grade 4 (*n* = 56). Demographic data are summarized in Table 1.

**Table 1:** Patient demographics

Variable	HCWHTO
Number of patients / knees	61 / 81
Gender (male / female), n (patients)	19 / 42
Gender (male / female), n (knees)	23 / 58
Preoperative age (years)	62.3 ± 7.0
BMI (kg / m²)	26.5 ± 3.3
Kellgren–Lawrence grading	
Grade 1, n	0
Grade 2, n	0
Grade 3, n	25
Grade 4, n	56

*BMI* Body mass index, *HCWHTO* Hybrid closed wedge high tibial osteotomy

### Clinical outcomes (2-year follow-up)

Clinical outcomes are summarized in Table 2. VAS scores significantly decreased postoperatively, and all J-KOOS subscale scores improved significantly compared with preoperative values (*p* < 0.001 for all comparisons). Range of motion showed a slight but statistically significant improvement in extension, whereas flexion remained unchanged. Preoperative flexion contractures were alleviated following HCWHTO. No major postoperative complications were observed.

**Table 2:** Pre and postoperative clinical evaluation

Variable	Preoperative	Postoperative	*p* value
VAS	4.1	1.1	< 0.001
Range of motion			
Extension (°)	-3.5 ± 4.7	-1.3 ± 3.1	< 0.001
Flexion (°)	130 ± 9.8	131 ± 10	0.52
J-KOOS subscales			
Symptoms (%)	57 ± 19	80 ± 14	< 0.001
Pain (%)	51 ± 17	77 ± 16	< 0.001
ADL (%)	65 ± 16	84 ± 14	< 0.001
Sport/Rec (%)	28 ± 20	56 ± 24	< 0.001
QOL (%)	31 ± 18	59 ± 24	< 0.001

*ADL* Activities of daily living, *J-KOOS* Japanese version of the Knee injury and Osteoarthritis Outcome Score, *QOL* Quality of life, *VAS* Visual analog scale

### Radiographic evaluation (2-year follow-up)

Radiographic outcomes are summarized in Table 3. Interobserver reliability was generally excellent (ICC > 0.90), although preoperative HKA angle demonstrated moderate agreement. Detailed ICC results are provided in Supplementary Table S1. Alignment parameters showed significant correction from varus to valgus, with a lateral shift in %MAD (*p* < 0.001). The Blackburne–Peel ratio increased slightly but significantly, and patellar tilt decreased significantly postoperatively (Table 3).

**Table 3:** Pre and postoperative radiographic evaluation

Variable	Preoperative	Postoperative	*p* value
HKA angle (°)	-8.2 ± 3.9	2.7 ± 3.4	< 0.001
%MAD (%)	7.1 ± 17.6	56.1 ± 15.0	< 0.001
Blackburn–Peel ratio	0.6 ± 0.3	0.7 ± 0.3	< 0.001
Patellar tilt (°)	4.9 ± 4.4	2.6 ± 3.0	< 0.001
Lateral patellar shift (mm)	5.1 ± 4.0	5.3 ± 4.0	0.53

*HKA* Hip–knee–ankle, *%MAD* Percentage mechanical axis deviation

### Arthroscopic outcomes (1-year follow-up)

#### Absolute evaluation (ICRS grade–based)

Because ICRS grades represent ordinal data, results are presented as median and interquartile range (IQR) (Table 4). Absolute ICRS grade–based evaluation of the PF joint revealed compartment-specific changes at 1-year follow-up. For the trochlear groove (primary structural outcome), the median ICRS grade was 3 (IQR 3–3) preoperatively and remained 3 (IQR 2–3) at one year. Although the median value was unchanged, the distribution shifted toward lower grades, with an increase in grade 2 lesions (16 to 26 knees) and a marked reduction in grade 4 lesions (14 to 3 knees). The Wilcoxon signed-rank test demonstrated a significant distributional shift (Z = − 3.10, *p* < 0.001), corresponding to a large effect size (*r* = 0.60). In the patellar facets, changes were more modest. The medial patellar facet showed a slight improvement in median ICRS grade from 3 (IQR 2–3) to 2 (IQR 2–3), whereas the lateral facet remained unchanged at 2 (IQR 2–3). Detailed changes in patellofemoral cartilage status across all PF compartments are presented in Table 5.

**Table 4:** Pre and postoperative ICRS grades

ICRS grade	Medial patellar facet		Lateral patellar facet		Trochlear groove		Medial tibial plateau		Medial femoral condyle		Lateral tibial plateau		Lateral femoral condyle	
	Pre	Post	Pre	Post	Pre	Post	Pre	Post	Pre	Post	Pre	Post	Pre	Post
0	0	0	0	0	0	0	0	0	0	0	0	1	8	7
1	5	6	5	7	0	0	0	1	0	0	15	13	60	57
2	35	47	42	46	16	26	2	5	2	6	54	55	9	12
3	36	25	27	25	51	52	11	28	10	19	11	10	4	3
4	5	3	7	3	14	3	68	47	69	56	1	2	0	2

*Pre* Preoperative, *Post* Postoperative, *ICRS* International Cartilage Repair Society

**Table 5:** Changes in ICRS grade in patellofemoral compartments

Compartment	Improved, *n* (%)	Unchanged, *n* (%)	Worsened, *n* (%)
Medial patellar facet	21 (25.9)	52 (64.2)	8 (9.9)
Lateral patellar facet	16 (19.8)	59 (72.8)	6 (7.4)
Trochlear groove	22 (27.2)	54 (66.7)	5 (6.2)

*ICRS* International Cartilage Repair Society

Spearman’s rank correlation analysis revealed no significant correlations between changes in any of the five J-KOOS subscales and changes in trochlear ICRS grade. In addition, no statistically significant differences were observed in J-KOOS subscale improvements between knees with and without macroscopic cartilage surface improvement (Table 6). In the tibiofemoral joint, absolute ICRS grade–based evaluation revealed predominantly medial compartment changes, while the lateral compartment showed no substantial alteration. In the medial femoral condyle, the median ICRS grade remained 4 (IQR 4–4 preoperatively and 3–4 at follow-up postoperatively). Similarly, in the medial tibial plateau, the median grade remained 4 (IQR 4–4 preoperatively and 3–4 postoperatively). In both compartments, the interquartile range shifted toward lower grades, reflecting a reduction in grade 4 lesions and a corresponding increase in grade 3 lesions at follow-up. In contrast, the lateral femoral condyle and lateral tibial plateau demonstrated no substantial change in median grade or IQR between preoperative and postoperative assessments.

**Table 6:** Association between changes in J-KOOS subscales and macroscopic arthroscopic cartilage surface improvement

J-KOOS subscale	ICRS change		Macroscopic cartilage surface improvement
	ρ	p value	p value
Pain	-0.19	0.10	0.32
Symptoms	-0.18	0.11	0.41
ADL	-0.16	0.15	0.28
Sport/Rec	-0.02	0.83	0.67
QOL	-0.05	0.68	0.54

*ADL* Activities of daily living, *ICRS* International Cartilage Repair Society, *J-KOOS* Japanese version of the Knee injury and Osteoarthritis Outcome Score, *QOL* Quality of life

#### Relative evaluation (macroscopic surface change–based)

**Fig. 4:**
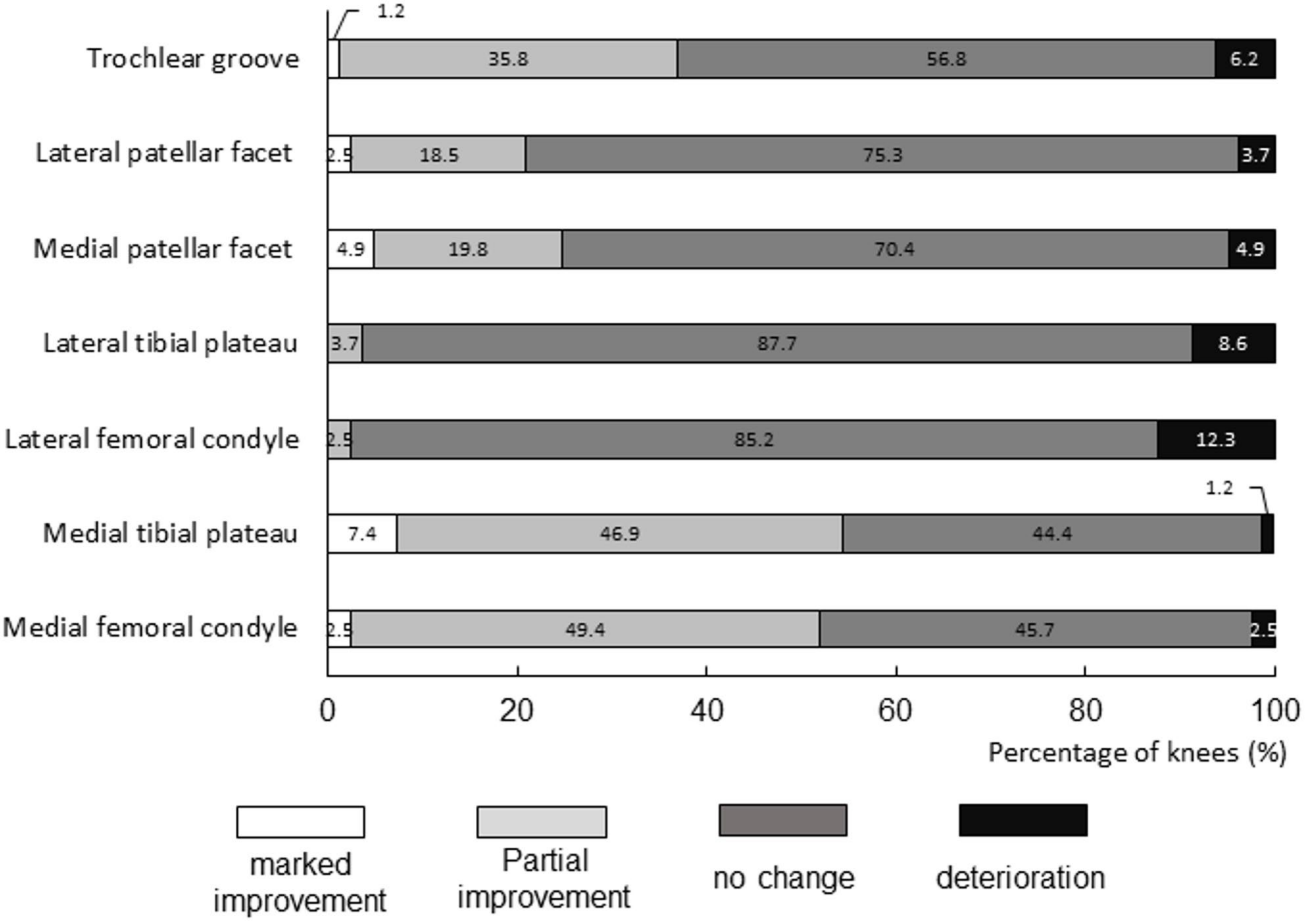
Distribution of 1-year macroscopic cartilage surface changes. Distribution of macroscopic cartilage surface changes in each compartment at 1-year second-look arthroscopy (81 knees). Percentages are presented for each category. Because of rounding, totals may not equal exactly 100%

**Fig. 5:**
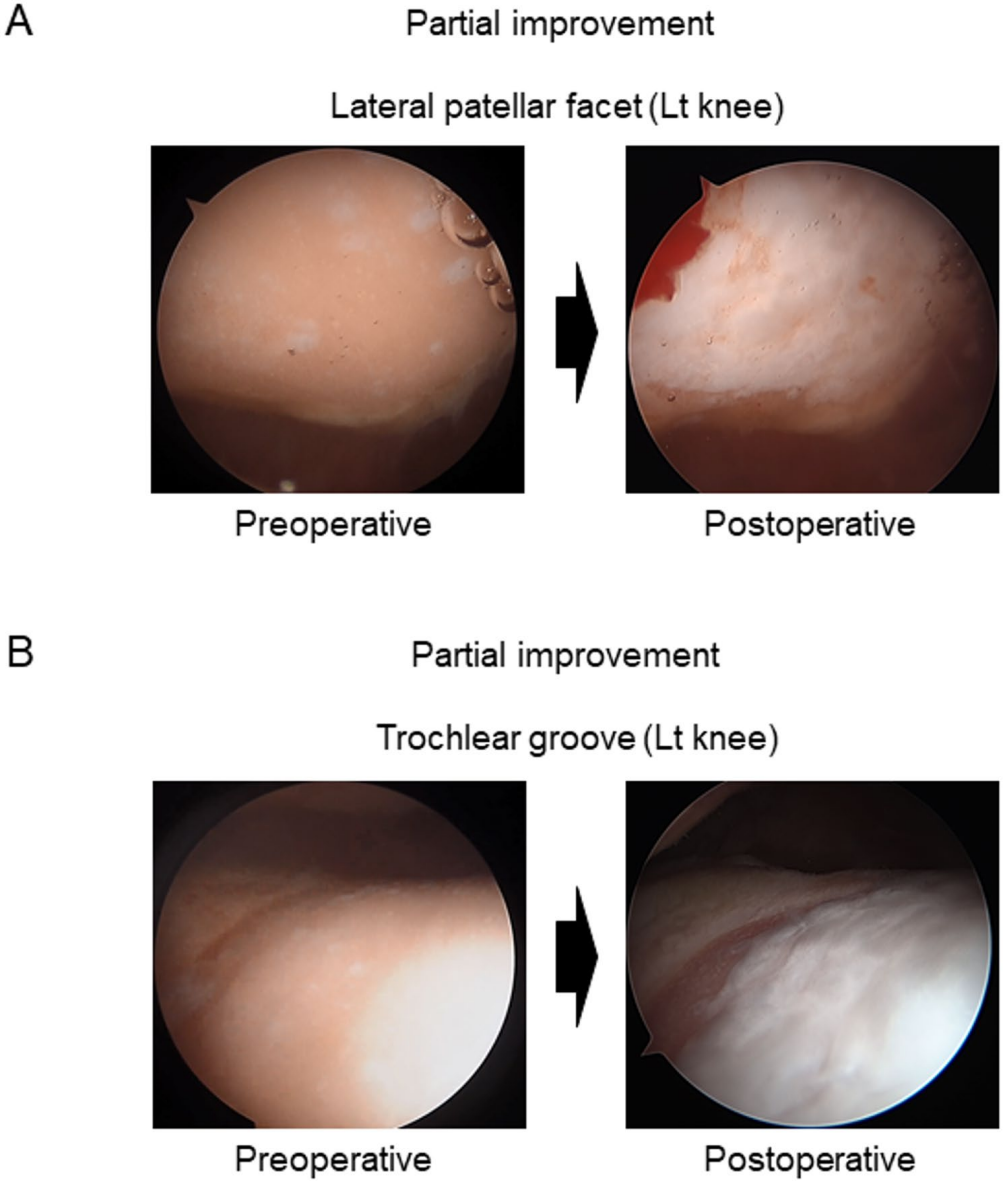
Representative patellofemoral joint changes. Representative arthroscopic findings of partial improvement in the patellofemoral joint at 1-year second-look arthroscopy. **A** Lateral patellar facet showing partial improvement with patchy repair-like tissue. **B** Trochlear groove showing partial improvement with broad but incomplete surface coverage

The distribution of 1-year macroscopic cartilage surface changes is shown in Fig. 4. Representative PF joint changes based on the 1-year relative evaluation are presented in Fig. 5. In the trochlear groove, marked or partial surface coverage consistent with improved tissue was observed in 30 of 81 knees (37.0%), including 1 knee (1.2%) with marked improvement and 29 knees (35.8%) with partial improvement, whereas deterioration was noted in 5 knees (6.2%). In the medial and lateral patellar facets, partial improvement was observed in 24.7% and 21.0% of knees, respectively, while deterioration was observed in less than 5% of cases in each region. In the lateral compartment, most knees demonstrated either partial improvement or no change. In the medial compartment, almost or partial improvement was observed in 48.1% of knees. No statistically significant differences were observed in changes in any of the five J-KOOS subscales between knees with and without macroscopic cartilage surface improvement (all *p* > 0.05; see Table 6).

## Discussion

The principal finding of this descriptive case series was that, at one year after HCWHTO, 37.0% of knees demonstrated macroscopic cartilage surface improvement in the trochlea, whereas deterioration was observed in 6.2%. Although the median ICRS grade remained unchanged at 3, the interquartile range shifted from 3 to 3 preoperatively to 2–3 postoperatively, accompanied by a marked reduction in grade 4 lesions. These findings suggest a redistribution of cartilage lesions toward lower grades rather than a uniform categorical improvement across the cohort. Importantly, most knees remained unchanged, and no significant association was observed between arthroscopic cartilage changes and improvements in J-KOOS subscales. Therefore, the present findings should be interpreted as short-term structural modifications rather than definitive evidence of cartilage regeneration or long-term clinical benefit. It should also be recognized that the reparative tissue observed arthroscopically is most likely fibrocartilage rather than native hyaline cartilage. Fibrocartilage is known to have inferior biomechanical properties and durability compared with hyaline cartilage. Therefore, even when macroscopic surface improvement is observed, its long-term structural integrity and clinical durability remain uncertain.

Previous studies have reported progression of PF cartilage degeneration following OWHTO. Kang et al. observed trochlear cartilage progression in 41.2% of knees at two-year arthroscopic follow-up [16], while Hanada et al. reported deterioration in 44% of knees [17]. Systematic reviews have similarly suggested that PF degeneration may occur after OWHTO, potentially associated with changes in patellar height and altered PF contact mechanics [4, 18]. In addition, long-term observational studies have demonstrated radiographic progression of PF osteoarthritis after OWHTO without necessarily correlating with worsened clinical outcomes [19, 20]. MRI-based investigations have also reported increased PF cartilage degenerative signals following OWHTO [21, 22]. In the present cohort, deterioration of trochlear cartilage was observed in 6.2% of knees at one-year follow-up. Although direct comparison is limited by differences in study design, follow-up duration, baseline PF status, and assessment methods, the short-term deterioration rate observed in this series was numerically lower than that reported in several OWHTO cohorts. Given the descriptive nature of the present study and the absence of a control group, these findings should be interpreted cautiously and do not permit conclusions regarding comparative effectiveness.

Akasaki et al. observed that DTO also demonstrated lower rates of PF cartilage deterioration than OWHTO [8]. Horikawa et al. demonstrated that DTO preserved patellar height and prevented progression of PFOA [11]. In their series, 10.9% of knees demonstrated improvement in ICRS grade of the femoral trochlea, 84.8% remained unchanged, and 4.3% showed progression at one-year second-look arthroscopy. In the present HCWHTO cohort, improvement in trochlear ICRS grade was observed in 27.2% of knees, whereas 66.7% remained unchanged and 6.2% worsened. Conventional CWHTO may also theoretically increase the Blackburne–Peel ratio through proximal migration of the tibial tuberosity, and therefore similar effects on PF mechanics might be expected. However, changes in patellar height alone do not consistently translate into favorable patellofemoral outcomes, and the relationship between patellar position and PF joint pressure remains contraoversial. In HCWHTO, in addition to proximal tuberosity migration, the biplanar osteotomy may induce internal rotation of the distal tibial fragment. Therefore, the observed redistribution of cartilage grades in the present study is unlikely to be explained by changes in the Blackburne–Peel index alone and is more likely multifactorial in nature.

Although differences in patient selection criteria and surgical indications preclude direct comparison, it should be noted that all included knees in the present study were classified as Kellgren–Lawrence grade 3 or 4, representing relatively advanced osteoarthritis. Cases requiring HCWHTO often involve larger correction angles compared with other osteotomy techniques. Therefore, differences in baseline disease severity and correction magnitude may limit direct comparison with studies evaluating other procedures, such as OWHTO or DTO, which are frequently performed in patients with less advanced deformity. Although differences in patient selection criteria and surgical indications preclude direct comparison, the proportion of knees demonstrating ICRS grade improvement was numerically higher in the present series. However, this observation should be interpreted cautiously given the differences in baseline disease severity and correction magnitude. These findings suggest that HCWHTO may be compatible with short-term stabilization of PF cartilage status in selected cases.

Biomechanically, PF contact mechanics are influenced by osteotomy configuration, sagittal slope control, and rotational alignment [6, 23–26]. Unlike OWHTO, HCWHTO involves complete medial cortical disruption and an oblique flange cut [2], which may induce proximal and anterior translation of the distal fragment as well as internal rotation [27]. In the present study, the postoperative increase in the Blackburne–Peel ratio and reduction in patellar tilt may reflect altered PF congruity. Stoffel et al. reported increased PF joint pressure following OWHTO but not after conventional CWHTO [7], supporting the hypothesis that osteotomy configuration may influence PF biomechanics. Nevertheless, the absence of correlation between arthroscopic findings and patient-reported outcomes suggests that mechanical unloading of the tibiofemoral joint remains the principal driver of symptomatic improvement after HTO [28–31]. Moreover, radiographic progression of PFOA does not consistently correlate with deterioration in clinical outcomes [19, 20], indicating that short-term structural modifications of the PF joint may not directly translate into measurable functional change.

Advanced knee osteoarthritis is frequently accompanied by PF involvement [7, 17, 32–34]. In such cases, joint-preserving surgical options remain limited. Although OWHTO and DTO may be appropriate for selected deformities, differences in correction magnitude and baseline PF pathology complicate direct comparison between techniques. Within these constraints, the present findings suggest that HCWHTO did not demonstrate marked short-term PF deterioration and was associated with redistribution toward lower ICRS grades in a subset of knees. However, careful patient selection and long-term follow-up are required to determine the durability and broader applicability of these observations.

### Limitations

First, this was a retrospective descriptive case series without a control group, and the findings should not be interpreted as evidence of comparative superiority. Second, follow-up duration was limited to one year for arthroscopic evaluation and approximately two years for clinical outcomes, precluding assessment of long-term durability. Third, arthroscopic assessment was performed at plate removal, which may have introduced selection bias. Fourth, cartilage evaluation was qualitative and based solely on macroscopic assessment without quantitative imaging or histological confirmation; therefore, the term “improvement” refers only to changes in surface appearance. Fifth, radiographic analysis was limited to two-dimensional measurements. Finally, reliability analysis was based on a limited subset of cases and intraobserver reliability was not assessed, which may affect generalizability.

## Conclusion

HCWHTO was associated with short-term macroscopic cartilage surface changes, characterized by redistribution toward lower ICRS grades in the trochlear cartilage in a subset of knees. However, these macroscopic cartilage surface changes were not associated with improvements in patient-reported outcomes. Given the absence of a control group and the limited follow-up duration, no conclusions can be drawn regarding comparative superiority or long-term protective effects.

## Supplementary Information

Supplementary Material 1.

## Data Availability

The datasets used and/or analyzed during the current study are available from the corresponding author on reasonable request.

## Abbreviations

ADL: Activities of daily living
CI: Confidence interval
CWHTO: Closed wedge high tibial osteotomy
DTO: Distal tuberosity osteotomy
HCWHTO: Hybrid closed wedge high tibial osteotomy
HKA: Hip–Knee–Ankle
HTO: High tibial osteotomy
ICC: Intraclass correlation coefficient
IQR: Interquartile range
J-KOOS: Japanese version of the Knee injury and Osteoarthritis Outcome Score
KL: Kellgren–Lawrence
MRI: Magnetic resonance imaging
OWHTO: Open-wedge high tibial osteotomy
PF: Patellofemoral
PFOA: Patellofemoral joint osteoarthritis
QOL: Quality of life
SD: Standard deviation
VAS: Visual analog scale
%MAD: Percentage Mechanical axis deviation
