# Supplementary material for: Hybrid closed wedge high tibial osteotomy is associated with short-term arthroscopic changes in the patellofemoral joint
Source: BMC Musculoskelet Disord. 2026 Apr 14;27:548. doi: 10.1186/s12891-026-09779-w (PMC13312685; doi:10.1186/s12891-026-09779-w)
Supplement: Supplementary file 1 — Supplementary Material 1. [file 12891_2026_9779_MOESM1_ESM.pdf]

Supplementary Table S1. Interobserver reliability (ICC [2,1]) of preoperative and postoperative radiographic measurements

Parameter	Preoperative ICC (95% CI)	Postoperative ICC (95% CI)	Combined ICC (95% CI)
HKA angle	0.57 (−0.01–0.88)	0.99 (0.96–1.00)	0.90 (0.77–0.96)
%MAD	0.99 (0.98–1.00)	0.99 (0.96–1.00)	1.00 (0.99–1.00)
Blackburne–Peel ratio	0.98 (0.92–1.00)	0.99 (0.96–1.00)	0.98 (0.96–0.99)
Patellar tilt	0.98 (0.92–1.00)	0.93 (0.73–0.98)	0.97 (0.92–0.99)
Lateral shift of patella	1.00 (0.99–1.00)	1.00 (0.99–1.00)	1.00 (0.99–1.00)

CI, confidence interval; HKA, hip–knee–ankle; ICC, intraclass correlation coefficient; %MAD, percentage mechanical axis deviation.
